# Effectiveness and Safety in Remote Monitoring of Patients with Pacemakers Five Years after an Implant: The Poniente Study

**DOI:** 10.3390/ijerph17041431

**Published:** 2020-02-23

**Authors:** Remedios López-Liria, Antonio López-Villegas, César Leal-Costa, Salvador Peiró, Emilio Robles-Musso, Rafael Bautista-Mesa, Patricia Rocamora-Pérez, Knut Tore Lappegård, Daniel Catalán-Matamoros

**Affiliations:** 1 Department of Nursing Science, Physiotherapy and Medicine, Hum-498 Research Team, Health Research Centre, University of Almería, 04120 Almería, Spain; liriareme@hotmail.com; 2 Social Involvement of Critical and Emergency Medicine, CTS-609 Research Team, Hospital de Poniente, 04700 Almeria, Spain; 3 Institute of Clinical Medicine. Faculty of Health Sciences, University of Tromsø, 9019 Tromsø. Norway; knut.tore.lappegard@gmail.com; 4 Nursing Department, University of Murcia, 30100 Murcia, Spain; cleal@um.es; 5 Health Services Research Unit, FISABIO-PUBLIC HEALTH, 04700 Valencia, Spain; peiro_bor@gva.es; 6 Intensive Care Unit, Hospital de Poniente, 04700 El Ejido-Almería, Spain; emiliomartin.robles-musso@ephpo.es; 7 Management Unit, Hospital de Poniente, 04700 Almeria, Spain; rafaeljesus.bautista@ephpo.es; 8 Division of Medicine, Nordland Hospital, N-8092 Bodø, Norway; 9 Department of Communication Studies, Universidad Carlos III de Madrid, 28903 Madrid, Spain; danieljcm79@hotmail.com; 10 Health Sciences CTS-451 Research Group, Health Research Centre, University of Almería, 04120 Almería, Spain

**Keywords:** health-related quality of life, long-term follow-up, remote monitoring, pacemaker, telemedicine

## Abstract

Health-related quality of life (HRQoL) and functional capacity values immediately after pacemaker (PM) implantation have been well established; however, not much has been known about its long-term effects. The present study compared the long-term effectiveness and safety of remote monitoring plus a clinic visit versus clinic visits alone during follow-up of adults implanted with PMs. This study was a single-centre, controlled, non-randomised, non-blinded clinical trial. Data were collected pre-implantation and after 60 months. The patients in the PONIENTE study were assigned to two different groups: remote monitoring (RM) and conventional monitoring (CM). The EuroQol-5D (EQ-5D) questionnaire was used to assess HRQoL and Duke Activity Status Index was used for the functional capacity. After five years, 55 patients completed the study (RM = 21; CM = 34). EuroQol-5D and functional capacity values were improved; however, significant differences were observed only in the EQ5D visual analogue scale (*p* < 0.001). Remote monitoring was equally feasible, reliable, safe, and clinically useful as CM. The frequencies of rehospitalisations and emergency visits did not differ between the groups. RM was found to be safe and effective in early detection and treatment of medical- and device-related events and in reducing hospital visits. Improved HRQoL was described not only immediately after PM implantation but also extended over a long time.

## 1. Introduction

Patients with an implanted pacemaker (PM) should undergo periodic check-ups to verify the accurate functioning of the device and to get it reprogrammed for optimal therapy, when necessary, to prevent repeated hospitalisations and predict the replacement time. The continuous adjustment of pacing parameters can afford important benefits to the patient and improve the effectiveness of treatment [1,2]. Regular follow-ups play a crucial role in the quick detection of technical and clinical faults that may appear during the functioning of the PM. However, it significantly increases the workload of the staff of institutions that manage these devices as evident from the fact that the most frequent task performed by cardiac electrophysiologists is to regularly check the implanted PMs [3,4]. Moreover, these patients often visit emergency hospitalisation and rehospitalisation with long periods of hospitalisation, resulting in reduced QoL [5].

In 2016, the European Society of Cardiology guidelines for the diagnosis and treatment of acute and chronic heart failure (CHF) recommended remote patient monitoring for those with implanted PM with a grade IIb and level of evidence B [6] and a goal to reduce the need for visits to the office/clinic. Previously, the class recommendation was IIa and the level of evidence A for device-based remote monitoring to facilitate early detection of atrial fibrillation, ventricular tachyarrhythmias and technical problems or issues (e.g., insulation defect, lead fracture, etc.) [7].

Remote monitoring (RM) or telemonitoring of patients with PMs is rapidly increasing although it was introduced just over ten years ago. RM is the vanguard of the personal ‘big data’ revolution in telemedicine or telehealth services [8]; it is necessary that we continue to improve the use of data generated by these devices to meet the needs and optimise the outcomes of patients as well as enable physicians to practice more efficiently [8,9]. Remote monitoring provides direct care at patients’ homes and to the people with chronic conditions, where it is used to improve patients’ feeling of safety and empower them to manage their condition and prevent hospitalisation [10,11,12]. RM of implanted cardiac devices allows the remote transfer of data stored in these devices from the patient’s home to a central database, where they are processed and shared with the treating physician or healthcare team. It does not provide any additional therapeutic capabilities to the device. The data transmission includes information stored in the device about arrhythmias, physiological parameters, device integrity, battery depletion, and lead failures that may result in inappropriate implantable cardioverter-defibrillator (ICD) shocks, an uncomfortable event that may increase the risk of life-threatening pro-arrhythmic events [13,14].

The systematic monitoring of the health-related quality of life (HRQoL) is being increasingly encouraged in chronic illness [15]. Several studies have provided data on HRQoL after PM implantation describing a clear improvement in HRQoL values evaluated after a few months [16,17] or one year [18]. Furthermore, it has been reported that remote cardiac monitoring provides increased freedom to patients and their family members. Their belief that the device will aid in the early detection of clinical or technical problems reduces the level of stress in their lives [13]. However, a study conducted on 1644 patients with cardiac implantable electronic devices (CIEDs) from seven European countries [19] demonstrated that most patients knew what device they were implanted with and felt sufficiently informed about the indications for therapy (90%). However, the majority of patients (83%) were followed up via face-to-face visits, because it was the most commonly preferred follow-up by them [19].

Increased expectations from telemonitoring have resulted in extensive experimentation with various telemonitoring services worldwide. For example, in Europe, especially in Italy, France, Germany or Spain, there have been numerous concluded experiments on CHF telemanagement or are still being performed [5].

In general, different trials have shown multiple advantages of RM; moreover, RM is not inferior to conventional follow-up in terms of safety in PMs [20]. Furthermore, RM decreases the time to make a clinical decision, thereby preventing several worsening conditions, allowing rapid detection of arrhythmias, reducing inappropriate shocks and sparing ICD batteries or device malfunctioning [4,21].

Moreover, it has been demonstrated that people need to be educated about the purpose of RM; the manner in which the information is transmitted, used and managed; and its benefits and limitations to provide information or perspectives on the ethical and social implications of technologies and treatments [13]. For instance, RM does not completely replace in-person clinic visits or is not intended as an emergency service. Some experts recommend an annual in-person evaluation after the first visit 2 to 12 weeks post-implantation, in addition to continuous RM plus remote interrogations every 3 to 12 months for PMs [22].

Therefore, more information on effectiveness would be very useful for National Health Services to determine if these new technologies could replace in-office visits and how they are been managed by professionals in every country.

We hypothesized that after discharge and in the long-term, RM of patients with pacemakers was at least as effective as clinic visits in terms to preserve/maintain HRQoL and functional capacity. The present study compared the long-term effectiveness and safety of RM plus a clinic visit versus clinic visits alone during follow-up of adults implanted with PMs.

## 2. Materials and Methods

### 2.1. Design

The present study was a long-term follow-up extension of a controlled, non-randomised, non-masked, single-centre clinical trial that compared RM with conventional monitoring (CM), with five years of follow-up from the date of PM implantation.

### 2.2. Setting and Patients

Between October 2012 and November 2013, a total of 89 patients were recruited in the Poniente Hospital (Almeria, Spain) for the original randomised controlled trial with a pre-planned follow-up of one year. Patient demographics, inclusion and exclusion criteria, operative details and outcomes have been reported previously [23,24].

Briefly, patients were included in the study if: (i) they had been implanted with a single [VVI-VVIR] or double chamber [VDD-DDD] Medtronic Carelink^®^ PM, (ii) they were at least 18 years old, (iii) they understand and correctly carried out the home auto-monitoring or a caregiver performed this action and (iv) they provided the signed informed consent to participate in the study. Patients were excluded if (i) they had been included in another study, (ii) they had any other cardiac device implanted or (iii) they did not agree to participate.

The study sample size was previously described in detail [23,24], estimated to be 90 patients (45 per group) in the main study variable (EQ-5D utilities) between the groups, assuming a standard deviation of 0.20, an alpha error of 0.05 and a power of 0.80.

### 2.3. Telecardiology System

The technology used in the study was the Medtronic CareLink^®^ Network, which is an internet-based RM service for patients with Medtronic implantable heart devices. The monitor gathered information from patients at home using a standard phone line and sent it to authorised hospitals [25]. Currently, PM communication with the transmitter is completely automatic in some models, without the intervention of the patient.

### 2.4. Procedure

One month following PM implantation, the cardiologist explained the advantages, disadvantages and characteristics of both monitoring modalities to the patients during a scheduled visit. If the patient selected the RM alternative, the cardiologist: (i) programmed the corresponding PM parameters; (ii) explained the use of the Medtronic CareLink monitor and the protocol for sending data to the patient; and (iii) requested the service from the supplier company. In accordance with the international guidelines [26,27], PM specifications, and physician’s criteria, the patients were asked to submit data at different times. In the RM group, follow-up visits were not scheduled. If the data received detected a cardiac event or a device dysfunction, the patients were contacted via phone and referred to a hospital visit. In the CM group, the patients had visits scheduled according to the cardiologist criterion and the standard practices of the Poniente Hospital. The first assessment was on the date of implantation and 5 years after implantation.

The Almería Health Research Ethical Committee approved the protocol and this was conducted in accordance with the precepts of the Declaration of Helsinki and Spanish laws on data protection and patient rights. Appropriate measures were considered to ensure data privacy after patients signed informed consent. The study was registered at ClinicalTrials.gov (Identifier: NCT02234245).

### 2.5. Measures and Instruments

The patient characteristics included age, gender, pacing indication (sinus node disease, atrioventricular block, others), disease manifestations (syncope, dizziness, dyspnea and angina), service derived (emergencies, cardiology, other services) and stimulation (VDD, DDD, VVI and VVIR).

Five years after PM implantation, differences between both the groups were assessed with regard to functional capacity, QoL, cardiovascular events (including episodes of paroxysmal atrial fibrillation (AF), AF episode durations, ischemic cerebrovascular events, anticoagulation, hospitalisation causes, hospitalisation days after PM implantation), and number of follow-up visits (transmissions from the hospital and patients’ home and total transmissions).

Duke Activity Status Index (DASI) was used to assess the functional capacity [28,29] (a Spanish validated version with punctuation from 33 [best state] to 11.5 [worst]).

EuroQol-5D (EQ-5D) is a Spanish-validated version which provides a subjective evaluation on the utility scores HRQoL [30,31] with punctuation from 0 [worst imaginable health state or death] to 100 [perfect health] and includes EQ-5D visual analogue scale (EQ-5D-VAS). These questionnaires were administered by means of personal and/or telephone interviews.

The research team revised the medical record for identifying cardiovascular events, changes in patient management and PM reprogramming at each scheduled visit and upon finish the study.

### 2.6. Statistical Analysis

First, the characteristics of the groups were compared using a difference in the means test for continuous variables and a difference in proportions test (binomial method) or Chi-Square test (replaced by Fisher’s exact test for cells with n < 5 cases) for qualitative variables. The differences between the groups in the pre-specified endpoints were also assessed using the difference in means or proportions tests. The corresponding 95% confidence intervals (95% CI) were included in the results. All analyses were performed using SPSS (SPSS Institute, Inc., Chicago, IL, USA) and STATA (College Station, TX, USA) statistical software.

## 3. Results

**Figure 1:**
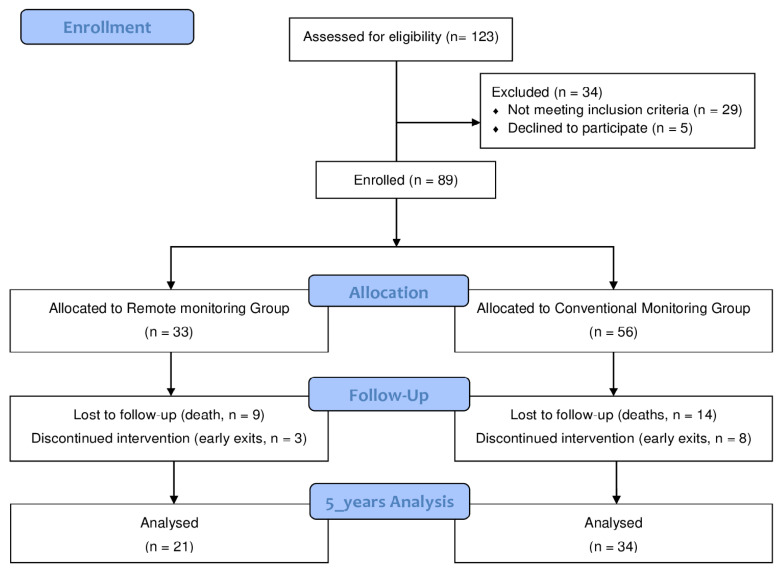
Flow (CONSORT) diagram of the study.

Five years after the PM implantation, 55 of the 89 initial patients (mean age: 81 ± 7 years, 31% women) finished the study (RM: 21 versus CM group: 34) in the PONIENTE trial (Figure 1). In general, syncope was the most prevalent and important disease manifestation (60%). The most frequent pacing indication was an atrioventricular block (71%), followed by sick sinus syndrome (20%). Dual-chamber PMs were used in 80% of the cases (VDD: 25.45% and DDD: 54.55%). While 47.27% of the patients reported experiencing paroxysmal AF episodes, only two of them (3.64%) had ischemic cerebrovascular events, although only 36.36% were on anticoagulation drugs. None of the assessed characteristics, including functional capacity and HRQoL, revealed significant differences between the groups (Table 1).

Of patients, 47% reported experiencing at least one episode of paroxysmal AF (RM: 67%, CM: 35%; *p* = 0.024) at the end of the study period (Table 1). Furthermore, three patients (5.46%) were hospitalised (RM: 2 versus CM: 1) because of causes without any direct relation to the pacing system. Twenty-two patients (RM: 8 vs. CM: 14) died of non-cardiovascular causes and six patients (RM: 1 vs. CM: 5) withdrew from the study.

A mean of 7.94 in-hospital visits per patient was registered in the CM group during follow-up, whereas a mean of 4.38 (*p* < 0.001) was reported in the RM group plus 6.62 home or remote transmissions.

### Health-Related Quality of Life and Functional Capacity

With respect to differences between the first evaluation and the end of the follow-up period (Table 2), every group reported significant improvements in the EQ-5D-VAS ratings (RM = +14.81, *p* < 0.001; CM = +14.86, *p* < 0.001). The EQ-5D utilities were increased in both groups (RM = +0.06, *p* = 0.48; CM = 0.10; *p* = 0.14) without significant differences. Increased functional capacity scores were reported only in the CM group (+0.79; *p* = 0.799).

There were no significant differences between the groups with respect to functional capacity or HRQoL. At five years, mean DASI score in the RM group was 20.48 and in the CM group was 20.74 (*p* = 0.84) (Table 3). On the EQ-5D-VAS, the RM group scored 73.81 and the CM group scored 72.94 (*p* = 0.88), whereas the respective scores were 0.68 and 0.77 on the EQ-5D utilities, without significant differences (*p* = 0.98).

## 4. Discussion

Both groups reported a similar distribution of demographic and clinical baseline characteristics including HRQoL, without any statistically significant differences. The mean age of patients was 81 years and one-third of the patients were women. This information is considerably similar to a recently published systematic review with six studies [13], where the mean age of the participants varied between 68 and 79 years and 45% to 65% were men. The indication for PM implantation in most cases was either atrioventricular block (66% to 89% in four studies), similar to 71% in the present study. Other indications included heart block, sinus node dysfunction and bundle branch block [20,32,33,34,35,36]. A recent study from Norway, with a similar design, described a mean age of 75 years with the most common pacing indication being sick sinus syndrome, followed by atrioventricular block and chronic atrial fibrillation with bradycardia [37]. Another study with CHF described mostly elderly patients in considerably good health and with little comorbidity [5].

An increase in HRQoL scores compared to baseline values was observed during the 5-year follow-up although a different course for separate scales was clear. The functional capacity scores were discreetly maintained or slightly declined at five years after implantation. These results are very similar to those of the Nordland study, where based on the Minnesota Living with Heart Failure questionnaire (MLHF) both follow-up groups showed statistically significant improvements in the baseline parameters, 12 months after the PM implantation. However, no significant differences were found in these parameters or any of the EQ-5D utility dimensions between the RM and CM groups at the enrolment and 12 months [37].

The PONIENTE study showed how the differences in terms of HRQoL or functional capacity were not statistically significant between the RM and CM groups and the number of in-office evaluations in the RM group was low. Despite a lower average number of in-office evaluations, patients receiving RM at home showed the same level of quality of life and functional capacity as the patients receiving follow-up in hospital, supporting our initial hypothesis. At the end of the study period, almost half of the patients reported experiencing at least one AF paroxysmal episode, without differences between the two groups. Three patients were hospitalised owing to the causes unrelated directly to the pacing system. Previous randomised trials have reported that RM was not inferior to conventional follow-ups in terms of safety when studying patients with PMs with different follow-up schemes [20,38]. The FOLLOWPACE study of HRQoL in 881 PM recipients described improvements using the PM-specific Aquarel questionnaire and the generic SF-36, observed at long-term [18]. As compared to men, they found lower long-term HRQoL on the overall SF-36 in women and worse long-term HRQoL at a higher age.

The findings in the present study showed no differences in the frequency of emergency visits or re-hospitalisations between the RM and CM groups at five years after PM implantation. There were no differences in the duration of hospital stays or use of emergency care clinic. Most of these findings have been documented in other studies with short follow-up such as a Southeast Asian, single-centre pilot study of 57 patients with CIEDs and RM via the Medtronic CareLink^®^ network [39]. This trial concluded that it was safe and feasible with follow-up for 6 months. Patients may prefer RM owing to possible improvements in the QoL. In the TARIFF study, the total number of in-hospital device follow-up visits was reduced by 58.78% in the RM group and the patients’ quality-adjusted life years were not significantly different between the groups [40].

The total number of clinic visits was statistically significantly lower in the RM group (4.38) versus the CM group (7.94). However, the number of unscheduled clinic visits was generally higher in the remote compared to the other group. In a systematic review, the mean total number of clinic visits per patient-year varied from 0.9 to 3.9 in the RM group and from 1.7 to 6.3 in the non-remote monitoring group within 12 to 37 months of follow-up [13]. Only Hindricks et al. (2014) did not show a decrease in the mean total number of clinic visits [41].

In terms of safety, other studies have shown that the majority of the routine in-office device followed in patients with home monitoring did not require intervention, as RM could accurately identify device malfunction or arrhythmias in patients [39]. Furthermore, the use of RM could offer more efficient healthcare delivery and possibly improve care standards, and it reduces the number of in-office visits [39,42].

Another study in line with these findings is the RM-ALONE [4], a multicentre prospective trial assigned 445 patients to evaluate the efficiency and safety of RM in patients with PMs and implantable cardiac defibrillators (ICDs) during 24 months. The study reported that RM reduced 79.2% the number of face-to-face visits, decreasing the workload of the personnel. Continuous ‘remote’ contact with patients sending clinical reports avoided unnecessary visits, often resulting from patient anxiety [4].

Four prospective randomised clinical studies, with a large number of CHF patients and a robust methodology reported positive results for the telemonitoring experience [43] or, at least, no significant differences in most variables between the intervention group and the standard care group and with punctual improvements in the QoL or physical functioning in the intervention group [44,45,46].

Koehler et al. (2018) reported, over the course of 12 months, that the RM group reduced the number of days lost due to unplanned cardiovascular hospital admissions [47]. For this TIM-HF2 care strategy, the key component was a well-structured tele-medical centre available 24 h a day to act promptly according to the individual patient’s risk profile (the staff included changes in the medication and admissions to the hospital as needed, in addition to educational activities).

The PONIENTE study had certain methodological limitations. First, this is a non-randomised study in which patients, advised by the physician (considering the sociodemographic characteristics of patients), could select the type of monitoring. As significant differences were not found between the two groups of follow-up in relation to patients’ characteristics at baseline and end of the study. However, the contextual effects of other essential variables for final outcomes were not detected. Nevertheless, the assignment form used in the present study was approximated to the daily practice and probably the outcomes would be closer to those expected in the real world than those obtained with randomisation (greater external validity versus less internal validity) [24,48,49]. This is an open trial in which both users with PMs and physicians knew the type of monitoring they were included in, an aspect that could potentially influence their behaviour. This is a single-centre study with a limited number of implants per year, the final size obtained reduced the real statistical power [50,51,52,53,54,55]. The small final sample size, derived from the single-centre study, might have not possibly detected significant statistical differences between the two groups, and the results could differ in other Health Centre Systems and social settings. Finally, regarding the generalisation of the results to other settings, the basal characteristics studied were considerably similar to those reported in the Spanish Pacemaker Registry [56], an aspect that supports this generalisation.

This study includes interesting results about the effect of patients RM compared to the patients following a standard outpatient visits regime, providing additional and comparatively more detailed information regarding different medical conditions such as chance of re-hospitalization and use of urgent clinic care. There are few longitudinal studies which have examined these aspects, given the advanced age of the study population (81 years), but long-term follow-up reveals the likewise long-term feasibility of pacemaker implantation in elderly patients. The lack of significant differences between the 2 groups, those with RM and those with CM, in terms of safety and effectiveness, reveals that institutions or clinics could switch to RM only, which can greatly reduce healthcare expenses (for both institutions and patients).

## 5. Conclusions

The PONIENTE study shows that RM plus a clinic visit works well compared to clinical follow-up alone. Remote monitoring resulted in a shorter time for the detection and treatment of medical- and device-related events, as well as fewer clinic visits without increasing the risk of cardiovascular events. HRQoL measurement with EQ5D showed an increase in HRQoL after PM implantation as well as five years later. During long-term follow-up, scores on the overall EQ5D utilities and DASI gradually declined in the RM group. However, the increased scores on EQ5D VAS subscale were retained during the long-term follow-up in the RM and CM groups.

The RM approach has proven to be safe and effective in reducing hospital visits. Increased HRQoL is observed not only immediately after PM implantation, but also in the long run. Remote monitoring was equally feasible, reliable, safe and clinically useful as CM and follow-ups. The frequencies of re-hospitalisations and emergency visits were not different between the groups.

## Figures and Tables

**Table 1:** Patient characteristics at five years after implant.

	All	Groups		*p*-Value
		Remote Monitoring	Conventional Monitoring	
Age (mean) ± SD	81.00 ± 6.47	81.14 ± 7.30	80.91 ± 6.01	0.690
Women (%)	17 (30.91)	8 (38.10)	9 (26.47)	0.365
DASI (mean) [95 CI]	19.46 [19.22; 21.76]	19.05 [16.42; 21.67]	19.72 [17.39; 22.05]	0.842
EQ5D utilities (mean) [95 CI]	0.73 [0.64; 0.83]	0.68 [0.50; 0.85]	0.77 [0.65; 0.90]	0.232
EQ5D VAS (mean)	73.27 [68.95; 77.60]	73.81 [67.46; 80.16]	72.9 [66.90; 78.99]	0.879
Pacing indication (%)				
Sinus node disease	11 (20.00)	3 (14.29)	8 (23.53)	0.493
Atrioventricular block	39 (70.91)	15 (71.43)	24 (70.59)	
Others	5 (9.09)	3 (14.29)	2 (5.88)	
Disease manifestations (%)				
Syncope	33 (60.00)	13 (61.90)	20 (58.82)	0.681
Dizziness	16 (29.09)	7 (33.33)	9 (26.47)	
Dyspnoea	3 (5.45)	0 (0)	3 (8.82)	
Angina	3 (5.45)	1 (4.76)	2 (5.88)	
**Service derived** (%)				
Emergencies	11 (20.00)	4 (19.05)	7 (20.59)	0.516
Cardiology	32 (58.18)	14 (66.67)	18 (52.94)	
Other service	12 (21.82)	3 (14.29)	9 (26.47)	
Stimulation (%)				
VDD	14 (25.45)	5 (23.81)	9 (26.47)	0.595
DDD	30 (54.55)	12 (57.14)	18 (52.94)	
VVI	8 (14.55)	4 (19.05)	4 (11.76)	
VVIR	3 (5.45)	0 (0)	3 (8.82)	
AF Paroxismal episodes (%)				
Yes	26 (47.27)	14 (66.67)	12 (35.29)	0.024
No	29 (52.73)	7 (33.33)	22 (64.71)	
AF episodes duration (mean) ± SD				
	2.62 ± 1.55	2.57 ± 1.65	2.67 ± 1.50	0.829
Ischemic cerebrovascular event (%)				
Yes	2 (3.64)	1 (4.76)	1 (2.94)	0.622
No	53 (96.36)	20 (95.24)	33 (97.06)	
Anticoagulation (%)				
Yes	20 (36.36)	11 (52.38)	9 (26.47)	0.052
No	35 (63.64)	10 (47.62)	25 (73.53)	
Hospitalisation causes (%)				
No hospitalisation	52 (94.55)	19 (90.48)	33 (97.06)	
Friedrich	1 (1.82)	1 (4.76)	0 (0.00)	0.323
PM electrode dislocation	1 (1.82)	1 (4.76)	0 (0.00)	
PM Fracture electrode	1 (1.82)	0 (0.00)	1 (2.94)	
Hospitalisation days after PM implantation (mean) ± SD				
	0.13 ± 0.70	0.95 ± 0.30	0.15 ± 0.86	0.322
Hospital visits (mean) ± SD				
	6.58 ± 2.74	4.38 ± 2.62	7.94 ± 1.79	<0.001
Home transmissions (mean) ± SD				
	2.53 ± 3.41	6.62 ± 1.72	---	
Total transmissions (mean) ± SD				
	9.11 ± 2.72	11 ± 2.93	7.94 ± 1.79	<0.001

N = 55 (RM group: 21; CM group: 34). 95 CI: 95% confidence interval of means or proportions. EQ5D: EuroQoL-5D; DASI: Duke Activity Status Index. SD: Standard Deviation; AF: Atrial Fibrillation; PM: Pacemakers; VAS: Visual Analogue Scale; Friedrich: Pacemaker wound infection.

**Table 2:** Differences in health-related quality of life and functional capacity at five years of follow-up in both groups.

	All				Remote Monitoring				Conventional Monitoring			
Functional Capacity												
	Baseline	Year 5	Differences	*p*-value	Baseline	Year 5	Differences	*p*-value	Baseline	Year 5	Differences	*p*-value
DASI [95CI]	20.49 [19.22; 21.76]	20.64 [18.84; 22.43]	0.15 [–1.33; 2.47]	0.548	21.42 [19.32; 23.52]	20.48 [17.63; 23.32]	−0.94 [−2,06; 4.01]	0.510	19.95 [18.32; 21.58]	20.74 [18.31; 23.16]	0.79 [−1.40; 4.79]	0.799
Health-Related Quality of Life												
EQ5D VAS [95CI]	58.41 [53.27; 63.56]	73.27 [68.95; 77.60]	14.86 [5.79; 20.21]	<0.001	59.00 [51.09; 66.91]	73.81 [67.46; 80.16]	14.81 [6.28; 29.91]	<0.01	58.08 [51.18; 75.33]	72.94 [66.90; 78.99]	14.86 [7.93; 29.13]	<0.001
EQ5D utilities [95CI]	0.70 [0.62; 0.78]	0.73 [0.64; 0.83]	0.03 [−0.11; 0.15]	0.745	0.74 [0.62; 0.86]	0.68 [0.50; 0.85]	−0.06 [−0.13; 0.27]	0.484	0.67 [0.56; 0.78]	0.77 [0.65; 0.90]	0.10 [−0.36; 0.06]	0.143

N = 55 (RM group: 21; CM group: 34). Values are expressed as means [95 CI: 95% confidence interval of means]. EQ5D: EuroQoL–5D; VAS: Visual Analogue Scale; DASI: Duke Activity Status Index; QALYs: Quality Adjusted Life Years.

**Table 3:** Differences between the groups regarding functional capacity and health-related quality of life at five years after pacemaker implantation.

	Remote Monitoring	Conventional Monitoring	*p*-Value
Functional Capacity			
DASI [95 CI]	20.48 [17.63; 23.32]	20.74 [18.31; 23.16]	0.84
Health-related Quality of Life			
EQ5D VAS [95 CI]	73.81 [67.46; 80.16]	72.94 [66.90; 78.99]	0.88
EQ5D utilities [95 CI]	0.68 [0.50; 0.85]	0.77 [0.65; 0.90]	0.98

N = 55 (TM group: 21; HM group: 34). Values are expressed as means [95 CI: 95% confidence interval of means]. EQ5D: EuroQoL-5D; VAS: Visual Analog Scale; DASI: Duke Activity Status Index.
